# X-chromosome association study reveals genetic susceptibility loci of nasopharyngeal carcinoma

**DOI:** 10.1186/s13293-019-0227-9

**Published:** 2019-03-25

**Authors:** Xiao-Yu Zuo, Qi-Sheng Feng, Jian Sun, Pan-Pan Wei, Yoon-Ming Chin, Yun-Miao Guo, Yun-Fei Xia, Bo Li, Xiao-Jun Xia, Wei-Hua Jia, Jian-Jun Liu, Alan Soo-Beng Khoo, Taisei Mushiroda, Ching-Ching Ng, Wen-Hui Su, Yi-Xin Zeng, Jin-Xin Bei

**Affiliations:** 1 State Key Laboratory of Oncology in South China, Collaborative Innovation Center for Cancer Medicine, Guangdong Key Laboratory of Nasopharyngeal Carcinoma Diagnosis and Therapy, Sun Yat-sen University Cancer Center, Guangzhou, 510060 People’s Republic of China; 2 Institute of Biological Sciences, Faculty of Science, University of Malaya, 50603 Kuala Lumpur, Malaysia; 3 Department of Biochemistry and Molecular Biology, Zhongshan School of Medicine, Sun Yat-Sen University, Guangzhou, 510080 People’s Republic of China; 4 RNA Biomedical Institute, Sun Yat-Sen Memorial Hospital, Sun Yat-Sen University, Guangzhou, 510120 People’s Republic of China; 5 Human Genetics, Genome Institute of Singapore, Agency for Science, Technology, and Research, Singapore, 138672 Singapore; 6 Molecular Pathology Unit, Cancer Research Centre, Institute for Medical Research, 50603 Kuala Lumpur, Malaysia; 7 Laboratory for International Alliance on Genomic Research, RIKEN Center for Integrative Medical Sciences, Yokohama, 230-0045 Japan; 8 Department of Biomedical Sciences, Graduate Institute of Biomedical Sciences, College of Medicine, Chang Gung Molecular Medicine Research Center, Chang Gung University, Taoyuan, 333 Taiwan; 9 Department of Otolaryngology, Chang Gung Memorial Hospital, Linkou, Taoyuan, 333 Taiwan; 10 Center for Precision Medicine, Sun Yat-sen University, Guangzhou, 510080 People’s Republic of China

**Keywords:** Nasopharyngeal carcinoma, Genetic susceptibility, X chromosome, Association study, Male predominance

## Abstract

**Background:**

The male predominance in the incidence of nasopharyngeal carcinoma (NPC) suggests the contribution of the X chromosome to the susceptibility of NPC. However, no X-linked susceptibility loci have been examined by genome-wide association studies (GWASs) for NPC by far.

**Methods:**

To understand the contribution of the X chromosome in NPC susceptibility, we conducted an X chromosome-wide association analysis on 1615 NPC patients and 1025 healthy controls of Guangdong Chinese, followed by two validation analyses in Taiwan Chinese (*n* = 562) and Malaysian Chinese (*n* = 716).

**Results:**

Firstly, the proportion of variance of X-linked loci over phenotypic variance was estimated in the discovery samples, which revealed that the phenotypic variance explained by X chromosome polymorphisms was estimated to be 12.63% (non-dosage compensation model) in males, as compared with 0.0001% in females. This suggested that the contribution of X chromosome to the genetic variance of NPC should not be neglected. Secondly, association analysis revealed that rs5927056 in *DMD* gene achieved X chromosome-wide association significance in the discovery sample (OR = 0.81, 95% CI 0.73–0.89, *P* = 1.49 × 10^−5^). Combined analysis revealed rs5927056 for *DMD* gene with suggestive significance (*P* = 9.44 × 10^−5^). Moreover, the female-specific association of rs5933886 in *ARHGAP6* gene (OR = 0.62, 95%CI: 0.47–0.81, *P* = 4.37 × 10^−4^) was successfully replicated in Taiwan Chinese (*P* = 1.64 × 10^−2^). rs5933886 also showed nominally significant gender × SNP interaction in both Guangdong (*P* = 6.25 × 10^−4^) and Taiwan datasets (*P* = 2.99 × 10^−2^).

**Conclusion:**

Our finding reveals new susceptibility loci at the X chromosome conferring risk of NPC and supports the value of including the X chromosome in large-scale association studies.

**Electronic supplementary material:**

The online version of this article (10.1186/s13293-019-0227-9) contains supplementary material, which is available to authorized users.

## Introduction

Nasopharyngeal carcinoma (NPC) is a human squamous-cell carcinoma that arises in the epithelium of the nasopharynx. The most remarkable feature of NPC is its restricted geographical distribution of incidence rate, which is about 30 per 100,000 in its prevalent regions including Southern China but rare in the western countries [1]. The etiology of NPC is a multifactorial process, involving genetic, viral, and environmental factors [2]. Large-scale case-control association studies and familial linkage studies have confirmed the genetic contribution to NPC predisposition, by revealing multiple susceptibility loci of NPC, such as HLA genes [3–6], *TNFRSF19* [5], *MECOM* [5], *GABBR1* [3], *XRCC3* [7], *ITGA9* [4], *TERT-CLPTM1L* [8–10], and *CIITA* [8]. However, these explain only a small fraction of the NPC heritability [11, 12].

The predominant incidence of males is another feature of NPC, with a male/female ratio of two to threefolds in diverse populations [13, 14]. The age-standardized rates of NPC for males:females were 21.8:8.1 per 100,000 person-years in Hong Kong [15] and 28.93:11.26 in Sihui, Guangdong, where both cities are NPC prevalent regions in Southern China [16]. Unequal exposures to environmental risk factors between males and females, such as smoking and diet habit, may partially contribute to the gender difference in the incidence of NPC [15]. It has been suggested that some tumor suppressor genes may contribute to gender bias in cancer development by escaping from the inactivation of the X chromosome in females [17]. Moreover, the involvement of the X chromosome has been implicated in the predisposition to NPC [18, 19]; however, the exact genetic components in the X chromosome have remained largely unexploited in NPC. By contrast, recent X chromosome association studies have identified novel risk loci at the sexual chromosome for follicular lymphoma [20], schizophrenia [21], systemic lupus erythematosus [22], etc., which partially explain the missing heritability of those complex diseases.

To explore the genetic contribution of the X chromosome to the gender difference of NPC incidence, we performed an X chromosome-wide association study, with the discovery data of 1615 cases and 1025 controls in Southern China, followed by two validation analyses involving a total of 543 cases and 735 controls from Taiwan Chinese and Malaysian Chinese, respectively.

## Methods

### Study subjects

The discovery sample included 1615 NPC cases and 1025 controls derived from a previous genome-wide association study (GWAS) in Southern Chinese [5]. All cases were recruited through Sun Yat-sen University Cancer Center (SYSUCC) in Southern China during October 2005 and October 2007, whereas the controls were recruited from several physical examination centers in local communities in Guangdong during the same period. As for the validation stage, two independent case-control cohorts of Chinese descendants were included, with 266 NPC cases and 450 controls recruited from different states of Malaysia [4, 23], and 277 NPC cases and 285 controls recruited from the northern parts of Taiwan, respectively [3]. The diagnosis of NPC was confirmed according to the World Health Organization (WHO) classification at each study site. The study was approved by the Institutional Review Board at SYSUCC. Informed consent was obtained from all participants. The information for all subjects is summarized in Additional file 1: Table S1.

### Genotyping and quality control

Individual genotypes had been determined as described previously [5]. In brief, the genomic DNA was extracted from peripheral blood sample using commercial (Qiagen) DNA extraction kit (Southern China and Taiwan studies) or conventional methods (Malaysia study), and the genotyping was conducted by using Illumina BeadChip arrays, according to the manufacturer’s protocols (Illumina, Inc., San Diego, CA, USA). For the discovery stage, quality checks were applied for each sample as described in the previous study [5], using autosomal SNPs and removing those with genotyping rate < 95%, excessive observed level of heterozygosity (departure from 3 standard deviation), cryptic relatedness, error or uncertainty in gender estimation, and population outliers through principal component analysis (PCA). A total of 1590 cases and 994 controls were retained for subsequent analyses. We extracted 18,133 genotyped SNPs at the X chromosome from the dataset and conducted further quality control filtering for the SNPs as suggested previously [24, 25]. As heterozygote of a SNP in a male should be deemed to be a genotyping/calling error, a fact of being haploidy for the X chromosome in a male, thus, we assigned a missing value for such a call in a male sample. The SNPs were removed if they met the following criteria [1]: with genotyping rate < 92% for all samples or call rate difference > 3% or *P* < 1 × 10^−5^ between gender [2], with the difference of heterozygosity level > 5% or *P* < 1 × 10^−3^ between cases and controls in males [3], with minor allele frequency (MAF) < 1% for both males and females [4], deviation from Hardy-Weinberg equilibrium test *P* < 1 × 10^−6^ in females, or [5] with heterozygote genotypes found in 5% of males (due to genotyping error). For SNPs in pseudo-autosomal regions (PAR), we used the same filtering criteria as for the autosomal SNPs in our previous study [5]. Finally, 6536 SNPs were removed, and 11,597 X chromosomal SNPs were used for subsequent analyses.

For the validation stage, genotypes were retrieved from the two GWAS studies [3, 4, 23]. Genotypes for unknown SNPs in the GWAS datasets were imputed by using non-PAR SNPs and IMPUTE2 program [26, 27], with 1000 Genomes Phase I integrated variant set (March 2012, Build 37) as the reference. Imputed SNPs with imputation score (INFO in the *.impute2_info output file) < 0.5 were considered low confidence and removed. Then, Gtool program (v0.7.5) was used to convert data from IMPUTE2 to PLINK. Genotypes with the threshold for calling genotypes (−threshold) < 0.9 were considered as low confidence and set as missing. Imputed SNPs were subjected to the same quality control procedure as that of the genotyped SNPs. As a result, 99,710 imputed loci were retained for downstream analysis. For the Malaysian Chinese dataset, 1000 Genomes Phase I interim set (June 2011, Build 37) was used as the reference. The genotypes of validating SNPs were retrieved from the Taiwan Chinese dataset as previously described [3].

### Statistical and bioinformatics analysis

Association tests were performed by using PLINK implemented with logistic regression analysis under the generalized linear model [28, 29]. Genotypes of the X chromosome loci were coded as [0, 2] for males and [0, 1, 2] for females, accounting for the random inactivation on females and assuming activation of one of the two X chromosomes in females. Given that a proportion of SNPs can escape from random inactivation, genotype coding of [0, 1] for males and [0, 1, 2] for females was also tested in combined samples to account for the escaping from random inactivation. Gender was treated as a covariate, and the first 10 principal components (PCs) were also adjusted in the association test in the discovery sample. To investigate whether there are sex-specific effects underlying X chromosome loci, we performed association tests for males and females separately and also tests accounting for gender × SNP interaction. For combined analyses of discovery and replication datasets, meta-analyses were conducted in R (version 3.2.3) using the “metafor” package (version 1.9–8). The heterogeneity among datasets was first evaluated by *I*^2^ and *Q* test. *I*^2^ > 50% or *P* < 0.05 were considered as heterogeneous, and the random-effect model was applied; otherwise, the fixed-effect model would be used.

We used linear mixed model approach implemented in GCTA (version 1.24.7) [30, 31] to estimate the contribution of the X chromosome on the proportion of the NPC phenotypic variance in the discovery dataset. The equal variance in both sexes (EV), full-dosage compensation (FDC), and non-dosage compensation (NDC) models were applied [32]. Only genotyped SNPs were used in this study to reduce the potential impact of subtle imputation uncertainty. For a comparison, we also estimated the variance explained by each autosome by using common SNPs passing QC and with MAF > 5%.

We used the simpleM method to address the effective number of independent association tests [33] since the Bonferroni correction for multiple testing was too conservative for the association test among loci with considerable linkage disequilibrium (LD). The simpleM method adopts a principal component analysis on the SNP correlation matrix to pick up the least “effective number” of tests accounting for ≥ 99.5% variance. In this study, the effective number was estimated as 2308, irrespective of genotyped or imputed loci. Therefore, we adopted the X chromosome-wide significance level as 0.05/2308 = 2.17 × 10^−5^. In the discovery stage, we chose a relatively relaxed significance threshold (*P* < 1 × 10^−3^) to select candidate SNPs for the follow-up replications, allowing inclusion of more SNPs and higher sensitivity of reproducible association signals. We used the R package “twoStageGwasPower” to calculate statistical power in this study, following the methods described previously [34]. The key raw data of this study have been uploaded onto the Research Data Deposit (RDD; http://www.researchdata.org.cn/; Number: RDDB2019000532).

Furthermore, noncoding susceptible SNPs identified in this study were subjected to HaploReg (V4.1; https://pubs.broadinstitute.org/mammals/haploreg/haploreg.php) [35] to annotate their functional and regulatory potentials on chromatin state and protein-binding annotations from the Roadmap Epigenomics and ENCODE projects, sequence conservation across mammals, and the effect of SNPs on regulatory motifs. Expression quantitative trait locus (eQTL) effects of the SNPs were estimated by using GTEx portal, where samples were collected from 53 non-diseased tissue sites across nearly 1000 individuals and tissue-specific gene expression and regulation with particularly the correlation between SNP and the expression of nearby genes was analyzed and archived (V7; https://gtexportal.org/home/).

## Results

### Genetic variance in X chromosome contributes to the risk of NPC

**Fig. 1:**
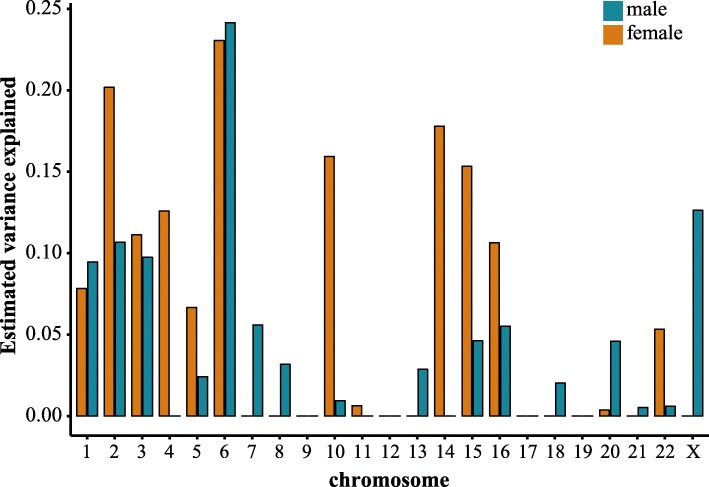
Estimates of the gender-specific genetic variance explained by autosomal and sexual chromosomes. The genetic variance of the X chromosome was estimated based on the equal variance (EV) model for females and non-dosage compensation (NDC) model for males. The genetic variance of autosomal chromosomes was estimated based on the EV model for both sexes

Firstly, the proportion of variance of X-linked loci over phenotypic variance was estimated under several dosage compensation models in the discovery samples for which the individual-level genotype data were available. X chromosome variants showed different contributions to the variance of NPC risk between males and females (Fig. 1). In males, 12.63% of the genetic variance estimated under non-dosage compensation (NDC) model was likely due to X chromosome variations (*P* = 0.024) and that was 6.74% and 3.49% under equal variance (EV) model (*P* = 0.024) and full-dosage compensation (FDC) model (*P* = 0.024), respectively. At whole genome level, the proportion ranked after that of chromosome 6, which contributed 24.14% of the genetic variance largely because of harboring the well-known risk loci of NPC as revealed by many GWAS studies [3–5](Additional file 1: Figure S1). However, as in females, X chromosome variants explained only 0.0001% variance for NPC under all three models, in contrast to the 23.04% of variance attributable to chromosome 6 (Fig. 1). These suggest that the contribution of the X chromosome to the genetic variance of NPC should not be neglected, hence motivating the search for loci associated with NPC in X chromosome.

### X chromosome-wide association analysis

**Fig. 2:**
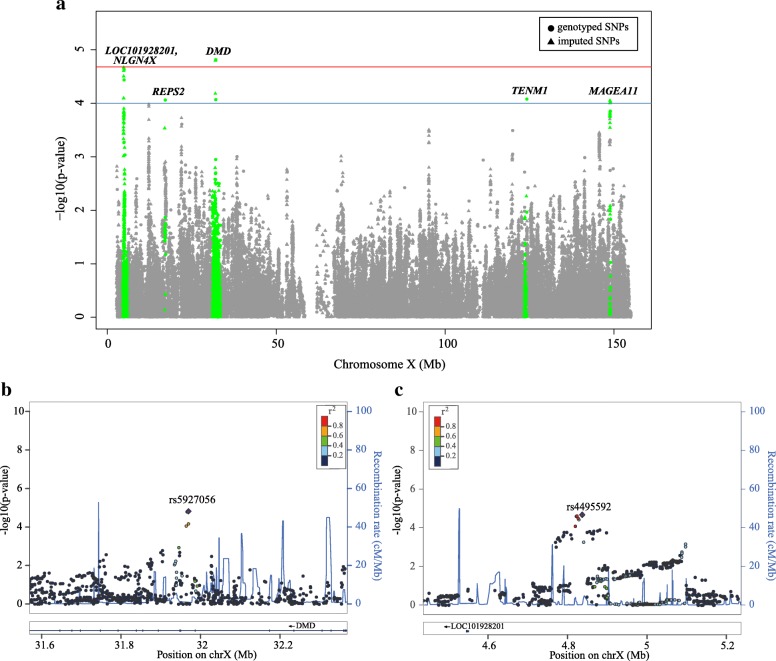
X chromosome-wide association of nasopharyngeal carcinoma. **a** Manhattan plot of *P* values for all X-linked SNPs (presented as −log_10_(*P*); circle and triangle for genotyped and imputed SNP, respectively). Steel blue line shows the suggestive significance level in the discovery study (*P* < × 10^−4^) and red line shows the X chromosome-wide significance (*P* < 2.17 × 10^−5^). **b** and **c.** Regional plot of *P* values from associations of rs5927056 and rs44955592, respectively, in the discovery dataset

Next, association tests were conducted for a total of 111,307 SNPs of X chromosome, including 11,597 directly genotyped and 99,710 imputed, in 1590 NPC cases and 994 controls. Significant associations surpassing the X chromosome-wide significance level (*P* = 2.17 × 10^−5^, Fig. 2a) were observed in two regions spanning *DMD* and *LOC101928201-NLGN4X*, where the leading SNPs were rs5927056 (OR = 0.81, 95% CI 0.73–0.89; *P* = 1.49 × 10^−5^, Fig. 2b) and rs4495592 (OR = 1.28, 95% CI 1.14–1.44; *P* = 2.15 × 10^−5^, Fig. 2c), respectively, together with additional supportive associations (Table 1 and Additional file 1: Table S2). Moreover, suggestive associations were observed in the other three loci, including *TENM1* (rs12842370), *REPS2* (rs12860876), and *MAGEA11* (rs2156978) (Additional file 1: Table S2; *P* < 1 × 10^−4^). Conditional analyses revealed that the sentinel SNPs accounted for all the associations observed in each of the five loci (Additional file 1: Figure S2). Given that a small portion of genes may escape from X chromosome inactivation, the non-random inactivation model was also applied in the association test. The association *P* values were highly consistent with those derived from random inactivation model (*r* = 0.85, Additional file 1: Figure S3) and an additional suggestive association was observed in *ARX-MAGEB18* (rs10127187, OR = 1.19, 95% CI 0.82–0.94; *P* = 8.04 × 10^−5^. Additional file 1: Table S3). Therefore, we adopted random inactivation model for the remaining analyses.

**Table 1:** Representative associations of SNPs on X chromosome for combined samples

			Discovery (Guangdong)			Replication 1 (Malaysia)			Replication 2 (Taiwan)			Combined analysis			
Marker	Position	Annotation	OR	95% CI	*P*	OR	95% CI	*P*	OR	95% CI	*P*	*I* ^2^	OR	95% CI	*P*
rs5927056	31969744	*DMD*	0.81	0.73–0.89	1.49 × 10^−5^	1.01	0.83–1.22	9.47 × 10^−1^	0.9	0.74–1.11	3.33 × 10^−1^	0.00	0.85	0.79–0.92	9.44 × 10^−5^
rs6641142	4825493	299 kb 3′ of *LOC101928201*	1.28	1.14–1.43	2.86 × 10^−5^	0.95	0.76–1.19	6.58 × 10^−1^	1.12	0.89–1.41	3.44 × 10^−1^	0.00	1.19	1.08–1.30	3.01 × 10^−4^
rs12860876	17037354	*REPS2*	1.20	1.09–1.31	7.20 × 10^−5^	0.95	0.81–1.12	5.58 × 10^−1^	1.01	0.85–1.21	8.86 × 10^−1^	68.81	1.07	0.92–1.23	3.98 × 10^−1^
rs2207942	145630047	261 kb 5′ of *CXorf51A*	1.18	1.08–1.29	4.02 × 10^−4^	1.14	0.96–1.36	1.43 × 10^−1^	0.96	0.79–1.17	7.04 × 10^−1^	0.00	1.14	1.06–1.23	7.30 × 10^−4^
rs2018094	12272097	*FRMPD4*	0.86	0.78–0.94	7.69 × 10^−4^	0.96	0.81–1.14	6.44 × 10^−1^	0.96	0.8–1.16	6.86 × 10^−1^	0.00	0.89	0.83–0.96	1.95 × 10^−3^
rs5910990	119872266	108 kb 3′ of *C1GALT1C1*	0.86	0.79–0.93	3.86 × 10^−4^	1.06	0.90–1.25	4.89 × 10^−1^	1.04	0.86–1.25	6.97 × 10^−1^	69.11	0.96	0.83–1.12	6.17 × 10^−1^
rs6528069	21915630	43 kb 5′ of *SMS*	0.85	0.78–0.93	2.41 × 10^−4^	1.11	0.94–1.31	2.07 × 10^−1^	1.11	0.92–1.34	2.76 × 10^−1^	80.29	1.00	0.83–1.21	9.86 × 10^−1^
rs5949698	95161542	430 kb 5′ of *BRDTP1*	1.22	1.10–1.37	3.40 × 10^−4^	0.86	0.70–1.05	1.39 × 10^−1^	0.91	0.73–1.12	3.74 × 10^−1^	80.91	1.00	0.79–1.26	9.90 × 10^−1^

**Fig. 3:**
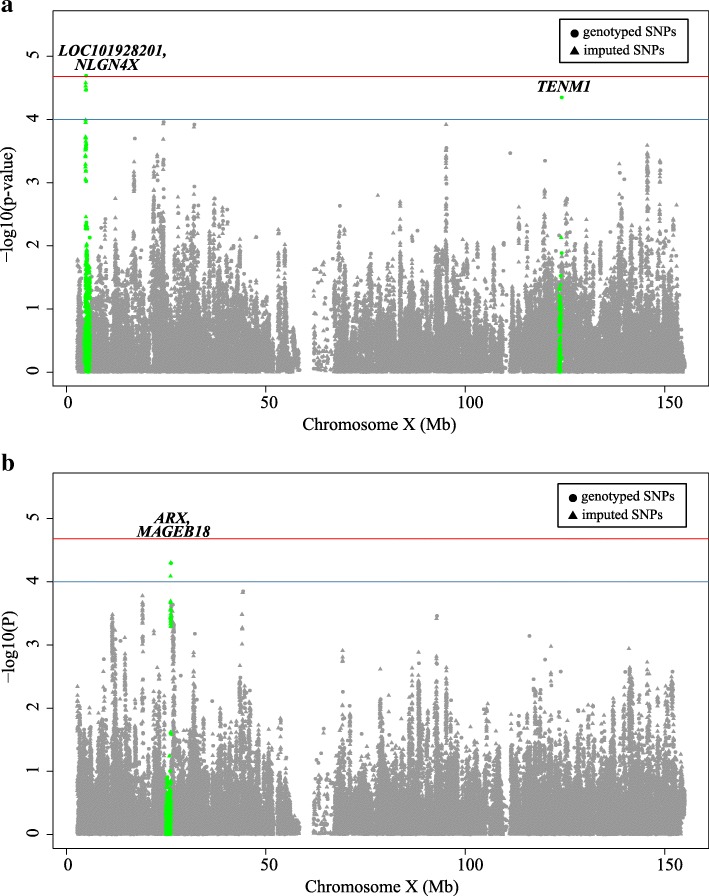
Gender-specific association of nasopharyngeal carcinoma. Manhattan plots of association results for **a** males and **b** females, respectively

Moreover, gender-specific association tests were carried out to identify X chromosomal loci that contribute to the sexual dimorphism phenomena in NPC. Suggestive gender-specific associations were found in the two genders, respectively (*P* < 1 × 10^−3^; Fig. 3 and Additional file 1: Figure S4). Sentinel signals were observed within the intergenic region of *LOC101928201*-*NLGN4X* (rs6641142, OR = 1.31, 95% CI 1.16–1.49, *P* = 2.50 × 10^−5^) and intron of *TENM1* (rs12842370, OR = 1.48, 95% CI 1.23–1.78, *P* = 4.51 × 10^−5^) for males (Fig. 3a) and 47Kb upstream of *MAGEB18* gene (rs10127187, OR = 0.42, 95% CI 0.29–0.65, *P* = 5.02 × 10^−5^) for females (Fig. 3b**)**. Some female-specific associations showed protective effects, such as the rs5933886 in *ARHGAP6* (OR = 0.62, 95%CI: 0.47–0.81, *P* = 4.37 × 10^−4^, Table 2). Gender-SNP interaction tests revealed several SNPs with nominal significance (*P* < 1 × 10^−3^), including rs2002686 in *EFHC2* (*P* = 8.4 × 10^−5^), rs139949129 and rs5933886 in *ARHGAP6* (*P* = 4.72 × 10^−4^ and 6.25 × 10^−4^, respectively), and some intergenic SNPs such as rs3859959, rs12834592, rs6603446, and rs72620283 (Additional file 1: Figure S5 and Table 2). Notably, after excluding the top SNPs (listed in Additional file 1: Tables S4 and S2 for males and all samples, respectively), the remaining variants explained less the genetic variances, with 1.67% under EV model in all samples, 4.63% under EV model and 8.85% under NDC model in males (Additional file 1: Figure S6).

**Table 2:** Representative gender-specific association on X chromosome in discovery cohort

Marker	Position	Minor allele	Annotation	MAF_males_^a^	OR_M_	*P*_M_	MAF_females_^a^	OR_F_	*P*_F_	OR_I_	*P*_I_
Males											
rs6641142	4825493	C	299 kb 3′ of *LOC101928201*	0.22/0.14	1.31	2.50 × 10^−5^	0.20/0.19	1.078	6.05 × 10^−1^	1.21	2.34 × 10^−1^
rs12842370	124071272	A	*TENM1*	0.47/0.44	1.22	4.51 × 10^−5^	0.51/0.47	1.05	7.02 × 10^−1^	1.15	2.58 × 10^−1^
rs6629842	24276842	C	427 kb 3′ of *ZFX*	0.49/0.40	1.21	1.21 × 10^−4^	0.47/0.50	0.87	2.17 × 10^−1^	1.35	1.20 × 10^−2^
rs5927056	31969744	G	*DMD*	0.22/0.3	0.81	1.17 × 10^−4^	0.22/0.27	0.77	4.66 × 10^−2^	1.04	7.62 × 10^−1^
rs140712668	95192068	T	400 kb 5′ of *BRDTP1*	0.39/0.31	1.22	1.20 × 10^−4^	0.35/0.38	0.85	1.77 × 10^−1^	0.89	1.20 × 10^−2^
Females											
rs10127187	26108742	C	47Kb 3′ of *MAGEB18*	0.06/0.08	0.87	8.65 × 10^−1^	0.05/0.11	0.42	5.02 × 10^−5^	2.02	2.40 × 10^−3^
rs10442369	44182366	A	*EFHC2*	0.32/0.28	1.08	1.40 × 10^−1^	0.24/0.33	0.63	1.42 × 10^−4^	1.66	1.19 × 10^−4^
rs6527885	19067421	A	*ADGRG2*	0.50/0.49	1.01	8.01 × 10^−1^	0.47/0.42	1.56	1.67 × 10^−4^	1.08	7.57 × 10^−2^
rs5933886	11463381	T	*ARHGAP6*	0.19/0.27	1.05	4.10 × 10^−1^	0.20/0.19	0.62	4.37 × 10^−4^	1.66	6.25 × 10^−4^
rs12847598	26558711	C	177Kb 5′ of *VENTXP1*	0.07/0.08	0.9	2.58 × 10^−1^	0.05/0.11	0.46	2.32 × 10^−4^	1.95	3.21 × 10^−3^
Gender-SNP											
rs2002686	44182139	A	*EFHC2*	0.31/0.27	1.08	1.29 × 10^−1^	0.23/0.33	0.62	1.48 × 10^−4^	1.69	8.35 × 10^−5^
rs3859959	43496280	C	20 kb 5′ of *MAOA*	0.39/0.45	0.88	7.88 × 10^−3^	0.47/0.38	1.42	2.12 × 10^−3^	0.63	1.22 × 10^−4^
rs12834592	92782494	C	143 kb 5′ of *NAP1L3*	0.44/0.47	0.94	2.00 × 10^−1^	0.49/0.39	1.51	3.49 × 10^−4^	0.63	1.86 × 10^−4^
rs6603446	115968398	G	374 kb 3′ of *CT83*	0.45/0.42	1.07	1.90 × 10^−1^	0.40/0.49	0.68	7.25 × 10^−4^	1.58	2.24 × 10^−4^
rs72620283	21923327	A	20 kb 3′ of *MBTPS2*	0.21/0.21	0.97	6.23 × 10^−1^	0.24/0.16	1.68	6.01 × 10^−4^	0.57	4.71 × 10^−4^
rs139949129	11449691	T	*ARHGAP6*	0.17/0.15	1.06	3.99 × 10^−1^	0.16/0.23	0.59	3.81 × 10^−4^	1.74	4.72 × 10^−4^

The association tests were conducted by using logistic regression adjusted for the first 10 principal components. *OR*_*M*_ and *P*_*M*_, odds ratio and *P* value for single point association test in males; *OR*_*F*_ and *P*_*F*_, odds ratio and *P* value for single point association test in females; *OR*_*I*_ and *P*_*I*_, odds ratio and *P* value for gender-SNP interaction

^a^MAF presents as minor allele frequency in cases/minor allele frequency in controls

### Validation and combined analyses

**Fig. 4:**
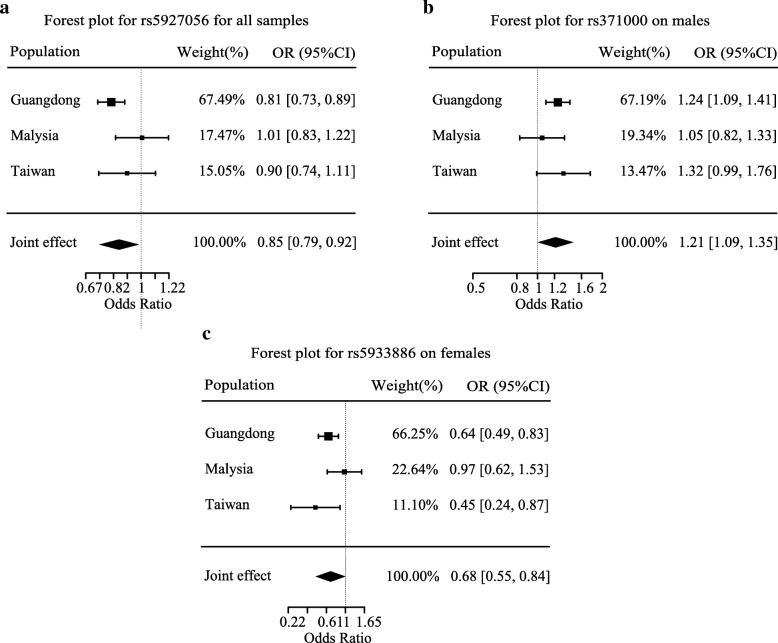
Forest plot of meta-analyses for X chromosome SNPs. Forest plot shows association results for **a** rs5927056 in all samples, **b** rs371000 in males, and **c** rs5933886 in females, respectively

A total of 27 independent candidate SNPs (pairwise *r*^2^ < 0.5) passing the suggestive significance level in discovery stage (*P* < 1 × 10^−3^) were selected for two independent follow-up validations in Taiwan Chinese and Malaysian Chinese, respectively. Three SNPs (rs12556646, rs12842370, and rs6540340) were excluded due to their absence in either of the validation datasets (Additional file 1: Table S5). Combined analyses suggested that rs5927056 in *DMD* gene were associated with NPC risk (*P* = 9.44 × 10^−5^; Fig. 4a). Moreover, combined analysis showed that rs371000 in *F9* (*P* = 3.13 × 10^−4^) was associated with NPC risk in males (Fig. 4b and Additional file 1: Table S4). For the female group, the combined analysis revealed that rs5933886 in *ARHGAP6* was associated with NPC risk (*P* = 2.05 × 10^−4^), and the association was validated in the Taiwan dataset (*P* = 1.64 × 10^−2^; Fig. 4c and Additional file 1: Table S6). Furthermore, the interaction of rs5933886 with gender was validated in the Taiwan dataset (*P* = 2.99 × 10^−2^). However, the associations within *REPS2* locus observed in the discovery sample were not significant in the combined analysis (Table 1).

### Functional annotations of the SNPs

In silico analyses were conducted to explore the functional potentials of the susceptibility SNPs on the X chromosomes. HaploReg revealed that the top significant rs5927056, located in the intron of *DMD*, might alter six regulatory motifs including AP-3, Evi-1_4, Hoxa10, Hoxb13, Hoxd10, and Pou2f2_known11, suggesting its regulatory potentials (Additional file 1: Table S7). HaploReg also revealed regulatory motifs (HMG-IY_1, Ik-3, NFKB_known5, Pou3f1, and STAT_known5) and DNase peaks at rs5933886, which is an intronic SNP of *ARHGAP6* (Additional file 1: Table S8). Moreover, the eQTL analysis revealed a significant *cis*-eQTL effect of rs5933886 in the aorta artery sample from the GTEx database, which were collected from the ascending aorta or other thoracic regions (nonatherosclerotic; *P* = 5.88 × 10^−3^, Additional file 1: Table S8). These results imply that rs5933886 may act as a regulatory SNP and predispose NPC by regulating the expression of *ARHGAP6*.

## Discussion

The human X chromosome consists ~ 155 million base pairs and contains more than 1000 genes that are important for many biological processes [17, 30]. However, none of the previous GWAS of NPC included association analysis of variations at the X chromosome, overlooking potential susceptibility loci that could partially account for the missing heritability of NPC. Here, we provided, to the best of our knowledge, the first large-scale X chromosome-wide association study of NPC in the Chinese population, revealing the contribution of the X chromosome and suggestive susceptibility loci for NPC risk.

At the chromosome-wide level, we observed different contribution of the X chromosome to NPC heritability between genders. The genetic variance of NPC could be partially explained by X chromosome for males, with considerable portion next to that by the chromosome 6 harboring the well-known susceptible HLA loci. However, a subtle genetic contribution of the X chromosome to NPC was observed in females. The different genetic effects on males and females might explain the sexual dimorphism phenomena in NPC incidence; however, the mechanisms underlying this phenomenon remain unclear. Random inactivation in the X chromosome is a major characteristic for females, such that homozygotes in females exhibit a similar genetic effect as hemizygotes in males. It has been implicated that X-linked tumor suppressor genes may escape from X-inactivation, whereby the females carrying such two copies of functional genes have a reduced risk to develop tumor [17]. Moreover, sex-specific regulatory variants are likely enriched in the X chromosome due to the sex-specific chromatin accessibility, implying different cumulative effects on gene reregulation between genders and thereby sex-biases in disease prevalence [36].

We identified a novel susceptibility locus at rs5927056 to be associated with NPC risk, surpassing X chromosome-wide significance in the discovery sample collection. Moreover, the combined analysis revealed a consistent association in males. Heterogeneous effect of the SNP was found in females (OR = 0.77, *P* = 0.04 in discovery data; OR = 0.99 and 1.00 in two replications), which could be partially explained by the inadequate statistical power because of limited female individuals included. Furthermore, the genetic variance of NPC risk to be explained by the X chromosome SNPs decreased with 8.3% after exclusion of rs5927056, suggesting a remarkable genetic effect of this locus on NPC susceptibility. rs5927056 is located within the intron of the *DMD* gene, a large gene that encodes the component of dystrophin-glycoprotein complex and bridges the inner cytoskeleton and the extracellular matrix. It has been demonstrated that *DMD* is sensitive to the replication stress on DNA damage and genome instability in tumor cells [37] and may act as a tumor suppressor involved in the development and progression of mesenchymal tumors [38]. Alteration in *DMD* was shown to be associated with many non-myogenic tumors [39]. Considering that rs5927056 alters some motifs in the region as revealed by HaploReg, we suspect that the haplotype of rs5927056 might regulate *DMD* expression and thus predispose individuals to NPC.

We also observed that rs5933886 in *ARHGAP6* was associated with NPC risk, specifically in females. *ARHGAP6* encodes Ras homology GTPase activation protein 6 and is involved in the regulation of actin polymerization at the plasma membrane during several cellular processes. ARHGAP6 may act as a tumor suppressor by inhibiting cell proliferation, migration, invasion, and adhesion of cervical carcinoma [40]. Our bioinformatics analyses revealed that rs5933886 was located within a regulatory region and was associated with *cis*-eQTL effects on *ARHGAP6*, suggesting that it may confer a protective effect on NPC risk for females through transcriptional regulation of *ARHGAP6*.

Although our study adopted a case-control approach with more than 3900 samples across multiple centers, the sample size is still our limitation. Only one third of the heritability on X chromosome could be explained by the identified association signals in current study. Given the observed minor allele frequency and effect size, the power of association tests in discovery dataset varied from 0.097 (rs371000 in males) to 0.841 (rs6418572 in females; Additional file 1: Table S9) under the present significance level (*α* = 2.17 × 10^−5^), suggesting that we had certain power to discover some susceptibility loci underlying the X chromosome and meanwhile other potential susceptibility loci would be overlooked under the current sample size setting. Moreover, as compared to that of the discovery cohort, the relatively smaller sample sizes of the replication cohorts might be insufficient to establish associations with effect size smaller than that in the discovery due to Winner’s curse phenomenon. Therefore, further studies with larger cohorts are necessary to boost the power for replicating our findings as well as detecting missing susceptibility loci on X chromosome, in particular for SNPs with low minor allele frequency and weak effect size.

Taken together, we reported for the first time the large-scale X chromosome-wide association study for NPC. Our study illustrates the value of association test by including the X chromosome in finding the missing heritability of complex diseases other than the conventional GWAS, particularly for those diseases showing the remarkable difference in incidence rate between genders. We acknowledge that future association studies are warranted to validate our findings in independent cohorts with a large sample size and to identify more novel genetic variants on sexual chromosomes associated with NPC risk. Moreover, fine-mapping studies incorporating next-generation sequencing technology would be important to pinpoint the causal variants underlying the susceptibility loci.

## Conclusion

Our study illustrates that the X chromosome confers different genetic effects on males and females and might explain the sexual dimorphism phenomena in NPC incidence. Our study reveals new susceptibility loci at the X chromosome conferring risk of NPC and supports the value of including the X chromosome in large-scale association studies. Further association studies using independent large cohorts are warranted to validate our findings.

## Additional file

Additional file 1: Supplemental materials. This file contains supplemental tables (Table S1-S8) and figures (Figure S1-S6). (PDF 2826 kb)
