# Supplementary material for: X-chromosome association study reveals genetic susceptibility loci of nasopharyngeal carcinoma
Source: Biol Sex Differ. 2019 Mar 25;10:13. doi: 10.1186/s13293-019-0227-9 (PMC6434801; doi:10.1186/s13293-019-0227-9)
Supplement: Supplementary file 1 — Supplemental materials. This file contains supplemental tables (Table S1-S8) and figures (Figure S1-S6). (PDF 2826 kb) [file 13293_2019_227_MOESM1_ESM.pdf]

Supplementary Figures and Tables

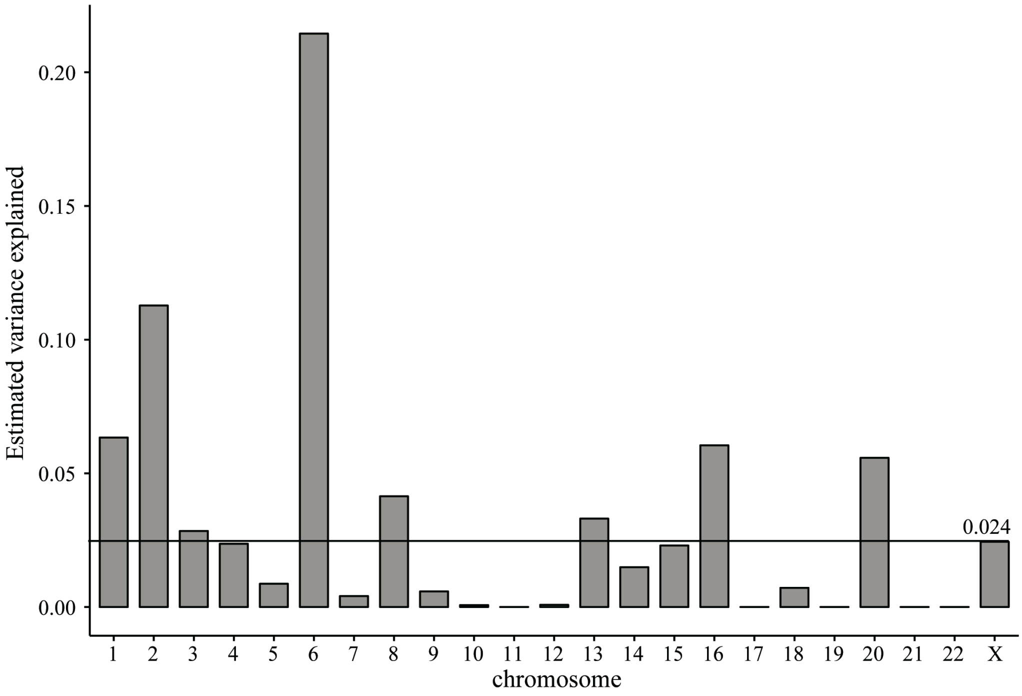

Figure S1. Estimates of the genetic variance explained by the X chromosome. The genetic variance of X chromosome was estimated based on the equal variance (EV) model.

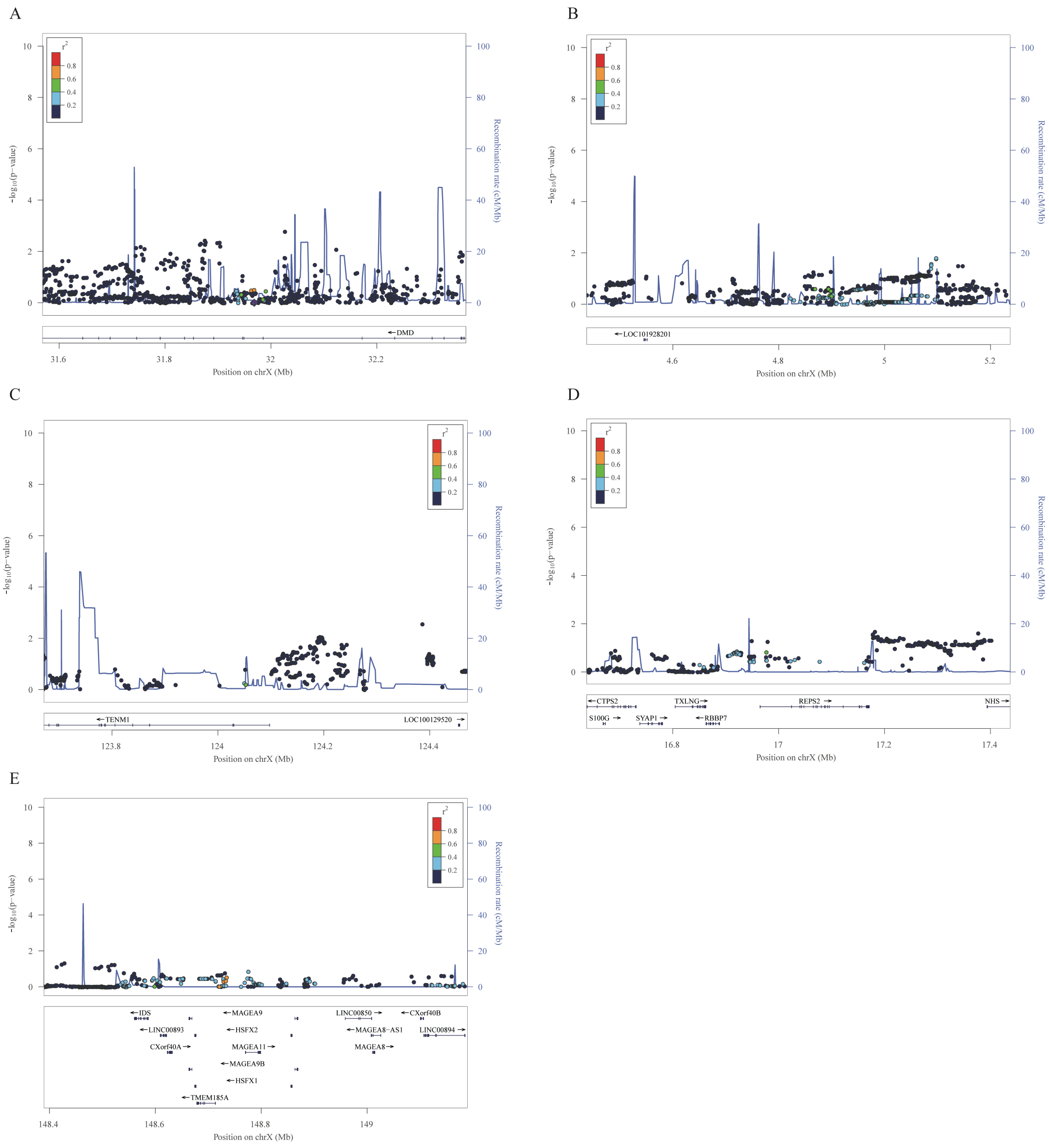

Figure S2. Conditional association analyses of the sentinel SNPs in X chromosome. Regional plot of *P* valuesfrom associations conditional on (A) rs5927056, (B)rs44955592, (C) rs12842370, and (D)rs2156978, respectively.

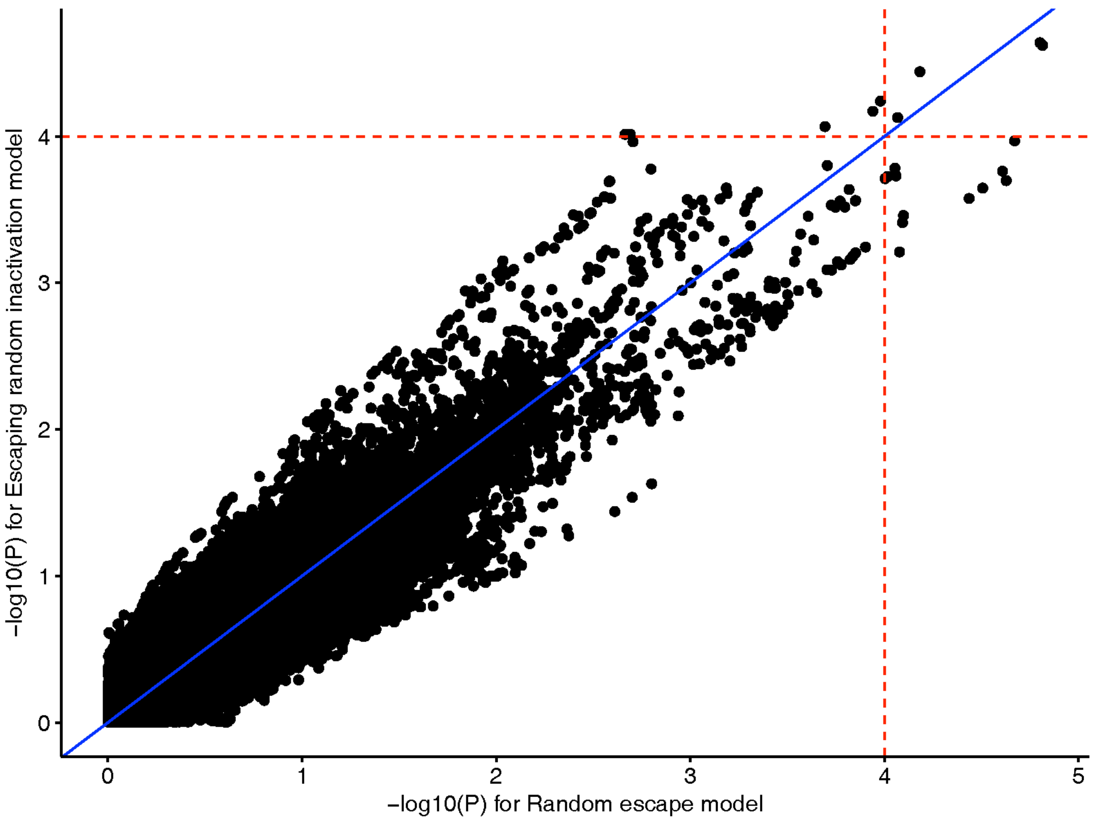

Figure S3. Comparison of the *P* values from different X-chromosome inactivation models on discovery cohort. The association tests were performed under either the random inactivation model or escaping random inactivation model, and the association results (presented as $-\log_{10}(P)$) were depicted as two-dimension scatter plot. Red dashed lines represent the suggestive significance level ($P<\times10^{-4}$) for both models, respectively. Blue solid line represents the diagonal line.

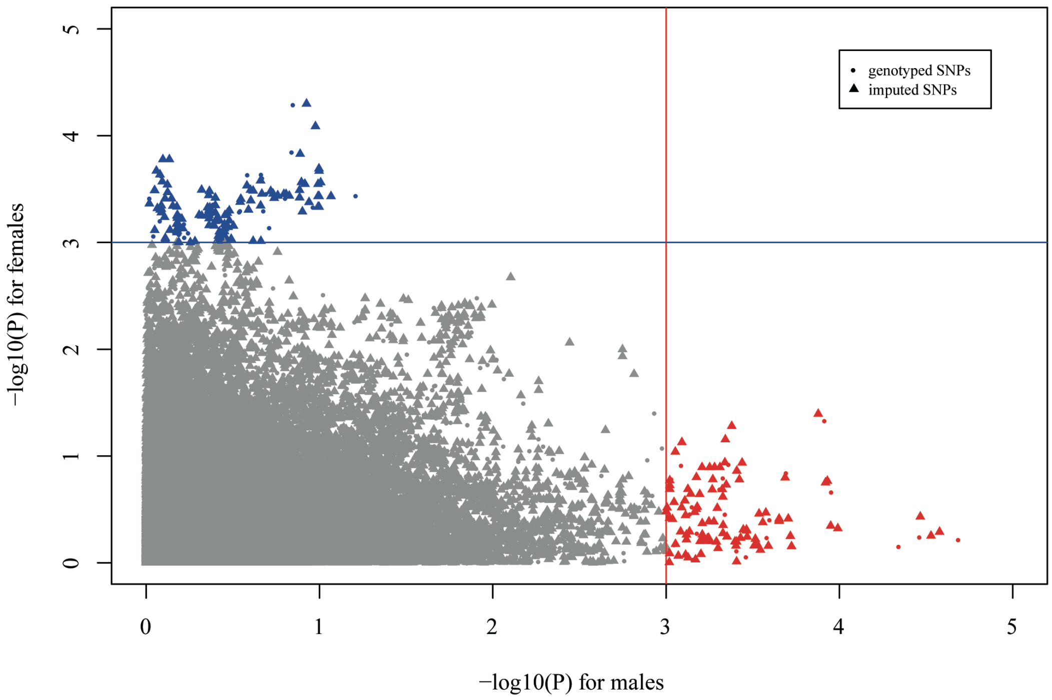

Figure S4. Comparison of the *P* values from associationin males and females. The association tests were performed separately for males and females, and the association results (presented as -$\log_{10}(P)$) were depicted as two-dimension scatter plot. Both genotyped (circle) and imputed (triangle) results were included. Red line and blue line represent the nominal significance level (P<×10^{-3}) for males and females, respectively. Nominally significant associated SNPs for males and females were in red and blue colors, respectively.

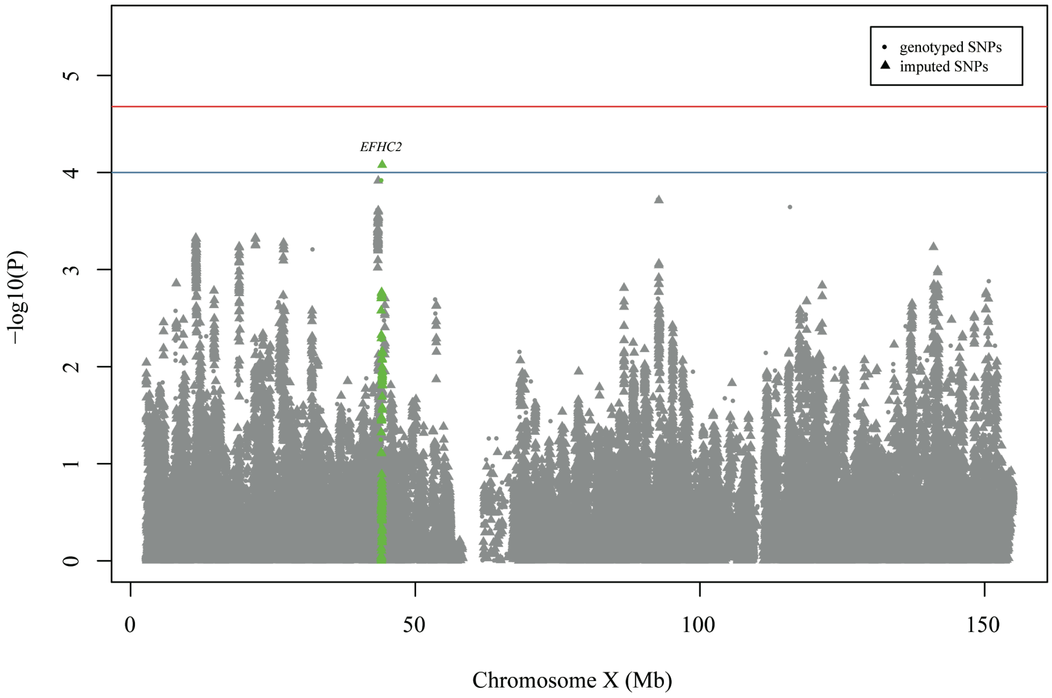

Figure S5. Manhattan plots of association results from SNP-gender interaction analysis.

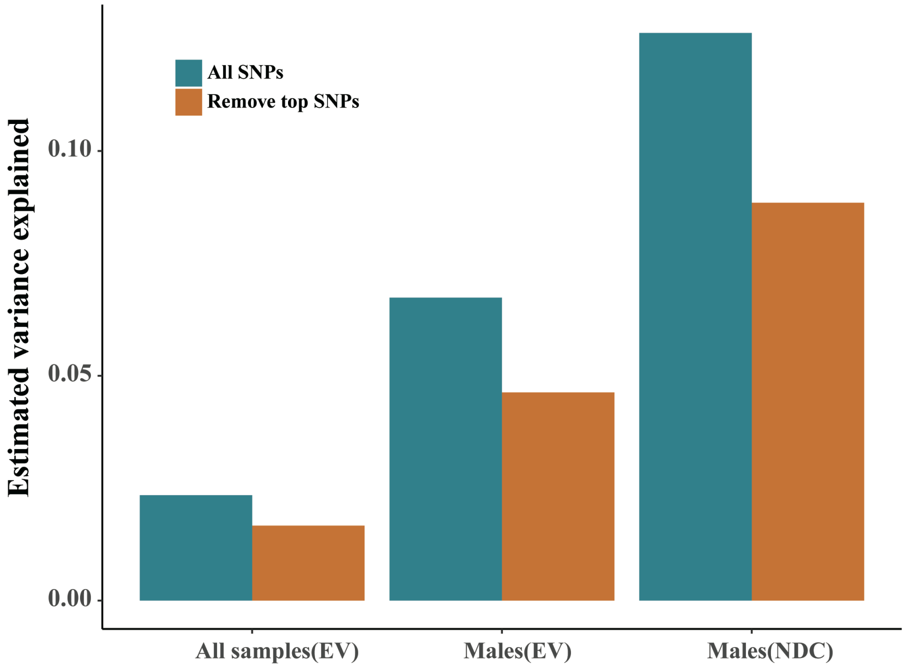

Figure S6. Change of genetic variance explained by X-chromosome variants after removing top significant SNPs.

Table S1. Sample characteristics of the datasets.

Stage	Population	Cases				controls			
		Sample size	Age(Mean ±SD)	Males	Females	Sample size	Age(Mean ±SD)	Males	Females
Discovery	Guangdong Chinese	1615	46.1 ±11.1	1182	433	1025	47.4±11.5	748	277
Replication1	Malasian Chinese	266	53.8±10.6	196	70	450	37.3±8.6	322	128
Replication2	Taiwan Chinese	277	48.6±11.9	210	67	285	50.4±13.0	192	93

Table S2. Top significant SNPs for the X chromosome association in combined samples in discovery cohort.

**Marker**	**Position**	**Minor allele**	**Annotation**	**MAF$_{cases}$**	**MAF$_{controls}$**	***OR***	**95% CI**	***P***
rs5927056	31969744	G	*DMD*	0.22	0.29	0.81	0.73-0.89	1.49×10^{-5}
rs4495592	4837130	A	299kb 3' of *LOC101928201*	0.22	0.16	1.28	1.14-1.44	2.15×10^{-5}
rs12842370	124071272	A	*TENM1*	0.49	0.55	1.19	1.10-1.30	8.40×10^{-5}
rs12860876	17037354	A	*REPS2*	0.45	0.38	1.19	1.09-1.30	8.75×10^{-5}
rs2156978	148789476	C	*MAGEA11*	0.26	0.32	0.83	0.75-0.91	8.87×10^{-5}

Logistic model adjusted for the first 10 principal components was conducted. Only the top significant SNPs in each locus were presented.

Table S3. Top significant SNPs for the X chromosome association under non-X-chromosome inactivation model in combined samples in discovery cohort.

**Marker**	**Position**	**Minor allele**	**Annotation**	***OR_NRI***	***P_NRI***	***OR_RI***	***P_RI***
rs6631437	31967612	G	*DMD*	0.92	2.31×10^{-5}	0.81	1.59×10^{-5}
rs12848742	12267153	A	*FRMPD4*	0.90	5.77×10^{-5}	0.77	1.05×10^{-4}
rs10127187	26108742	C	*47kb 5'* of *MAGEB18*	1.19	8.40×10^{-5}	0.77	2.05×10^{-3}

OR_RI and *P_RI*: Odds ratio and *P* value of SNP assuming random inactivation.

OR_NRI and *P_NRI*: Odds ratio and *P* value of SNP assuming escaping from random inactivation.

Logistic model adjusted for the first 10 principal components was conducted. Only the top significant SNPs in each locus were presented. Only loci with P-value of the top significant SNPs < 1×10^{-4} were shown.

Table S4. Replication and combined analyses of SNPs on X chromosome for males.

			Discovery (Guangdong)			Replication 1 (Malaysia)			Replication 2 (Taiwan)			Combined analysis			
Marker	Position	Annotation	*OR*	95% CI	*P*	*OR*	95% CI	*P*	*OR*	95% CI	*P*	I^2	*OR*	95% CI	*P*
rs2207942	145630047	261kb 5' of *CXorf51A*	1.19	1.08-1.32	5.92×10^{-4}	1.20	0.99-1.45	5.85×10^{-1}	1.08	0.87-1.33	5.10×10^{-1}	0.00	1.18	1.08-1.28	1.03×10^{-4}
rs371000	138615521	*F9*	1.25	1.10-1.42	8.37×10^{-4}	1.05	0.83-1.33	6.94×10^{-1}	1.32	0.99-1.76	5.89×10^{-2}	0.00	1.22	1.09-1.35	3.13×10^{-4}
rs6641142	4825493	299kb 3' of *LOC101928201*	1.31	1.16-1.49	2.50×10^{-5}	0.96	0.75-1.22	7.29×10^{-1}	1.09	0.85-1.4	5.12×10^{-1}	0.00	1.20	1.09-1.33	3.88×10^{-4}
rs5927056	31969744	*DMD*	0.81	0.73-0.90	1.17×10^{-4}	1.01	0.82-1.25	9.22×10^{-1}	0.89	0.71-1.11	2.94×10^{-1}	0.00	0.85	0.78-0.93	4.15×10^{-4}
rs6629842	24276842	42kb 3' of *ZFX*	1.20	1.09-1.32	1.33×10^{-4}	1.01	0.84-1.20	9.26×10^{-1}	1.03	0.84-1.25	7.88×10^{-1}	0.00	1.14	1.05-1.23	1.18×10^{-3}
rs4484871	22751065	*PTCHD1-AS*	0.78	0.68-0.90	4.57×10^{-4}	0.92	0.69-1.24	5.95×10^{-1}	1.01	0.72-1.41	9.66×10^{-1}	0.00	0.83	0.73-0.93	1.60×10^{-3}
rs1323329	139741667	50kb 3' of *LINC00632*	0.85	0.78-0.94	8.72×10^{-4}	1.00	0.83-1.20	9.80×10^{-1}	1.02	0.83-1.24	8.77×10^{-1}	0.00	0.90	0.83-0.97	7.39×10^{-3}
rs12860876	17037354	*REPS2*	1.20	1.09-1.33	1.65×10^{-4}	0.97	0.81-1.16	7.64×10^{-1}	0.98	0.81-1.2	8.56×10^{-1}	65.38	1.07	0.92-1.24	3.96×10^{-1}
rs5949698	95161542	430kb 5' of *BRDTP1*	1.25	1.11-1.41	2.86×10^{-4}	0.86	0.69-1.07	1.86×10^{-1}	0.96	0.76-1.2	7.02×10^{-1}	77.94	1.03	0.81-1.29	8.23×10^{-1}
rs5910990	119872266	108kb 3' of *C1GALT1C1*	0.85	0.77-0.93	5.42×10^{-4}	1.08	0.91-1.30	3.78×10^{-1}	1.04	0.85-1.26	7.20×10^{-1}	70.34	0.97	0.82-1.14	6.85×10^{-1}
rs6528069	21915630	43kb 5' of *SMS*	0.85	0.77-0.94	7.94×10^{-4}	1.12	0.94-1.34	2.17×10^{-1}	1.16	0.95-1.43	1.43×10^{-1}	80.65	1.02	0.83-1.25	8.57×10^{-1}

Table S5. List of the 27 independent candidate SNPs selected for validations.

Marker	Position	Annotation	Candidate association	Note
rs6641142	4825493	299kb 3' of *LOC101928201*	all, male	
rs5933886	11463381	*ARHGAP6*	female	
rs5935253	12195747	*FRMPD4*	female	
rs2018094	12272097	*FRMPD4*	all, male	
rs5935567	13442669	82kb 3' of *LINC01203*	female	
rs12860876	17037354	*REPS2*	all, male	
rs6633192	19067926	*ADGRG2*	female	
rs6528069	21915630	43kb 5' of *SMS*	all, male	
rs4484871	22751065	*PTCHD1-AS*	male	
rs6629842	24276842	42kb 3' of *ZFX*	male	
rs6418572	26109128	47kb 5' of *MAGEB18*	female	
rs12847598	26558711	143kb 5' of *NAP1L3*	female	
rs4495593	26873542	294kb 3' of *VENTXP1*	female	
rs5927056	31969744	*DMD*	all, male	
rs5972485	32091819	*DMD*	female	
rs5906154	43493189	21kb 5’ of *MAOA*	interaction	
rs10442369	44182366	*EFHC2*	female	
rs12834592	92782494	143kb 5' of *NAP1L3*	female	
rs5949698	95161542	430kb 5' of *BRDTP1*	all, male	
rs12556646	111152042	*TRPC5*	all	absence in Malaysia dataset
rs6603446	115968398	374kb 3' of *CT83*	female	
rs5910990	119872266	108kb 3' of *C1GALT1C1*	all, male	
rs12842370	124071272	*TENM1*	all, male	absence in Malaysia dataset
rs371000	138615521	*F9*	male	
rs1323329	139741667	50kb 3' of *LINC00632*	male	
rs2207942	145630047	261kb 5' of *CXorf51A*	all, male	
rs6540340	148796588	*MAGEA11*	all	absence in Taiwan dataset

Table S6. Replication and combined analyses of SNPs on X chromosome for females.

			Discovery (Guangdong)			Replication 1 (Malaysia)			Replication 2 (Taiwan)			Combined analysis			
Marker	Position	Annotation	*OR*	95% CI	*P*	*OR*	95% CI	*P*	*OR*	95% CI	*P*	I^2	*OR*	95% CI	*P*
rs5933886	11463381	*ARHGAP6*	0.62	0.47-0.81	4.37×10^{-4}	0.97	0.62-1.53	9.04×10^{-1}	0.45	0.24-0.87	1.64×10^{-2}	0.00	0.66	0.53-0.82	2.05×10^{-4}
rs6633192	19067926	*ADGRG2*	1.54	1.22-1.94	2.37×10^{-4}	1.01	0.68-1.51	9.51×10^{-1}	1.39	0.88-2.22	1.61×10^{-1}	0.00	1.39	1.16-1.67	4.46×10^{-4}
rs5935567	13442669	82kb 3' of *LINC01203*	0.59	0.43-0.80	8.68×10^{-4}	0.82	0.46-1.45	4.92×10^{-1}	0.86	0.46-1.6	6.35×10^{-1}	0.00	0.67	0.52-0.86	1.52×10^{-3}
rs5972485	32091819	*DMD*	0.60	0.45-0.81	6.68×10^{-4}	1.20	0.63-2.30	5.74×10^{-1}	1.02	0.56-1.87	9.45×10^{-1}	0.00	0.72	0.57-0.92	9.35×10^{-3}
rs5935253	12195747	*FRMPD4*	0.66	0.51-0.84	8.12×10^{-4}	1.09	0.67-1.77	7.20×10^{-1}	1.03	0.62-1.68	9.23×10^{-1}	0.00	0.77	0.63-0.94	1.11×10^{-2}
rs10442369	44182366	*EFHC2*	0.63	0.49-0.80	1.42×10^{-4}	1.09	0.72-1.65	7.00×10^{-1}	1.12	0.70-1.82	6.35×10^{-1}	71.42	0.88	0.59-1.31	5.26×10^{-1}
rs12847598	26558711	17kb 5' of *VENTXP1*	0.46	0.30-0.70	2.32×10^{-4}	2.36	1.17-4.74	1.62×10^{-2}	2.32	1.00-5.39	5.01×10^{-2}	88.85	1.30	0.43-3.92	6.39×10^{-1}
rs12834592	92782494	143kb 5' of *NAP1L3*	1.51	1.20-1.89	3.49×10^{-4}	1.19	0.80-1.76	4.03×10^{-1}	0.70	0.45-1.08	1.09×10^{-1}	79.07	1.11	0.71-1.72	6.46×10^{-1}
rs4495593	26873542	294kb 3' of *VENTXP1*	0.65	0.51-0.83	6.27×10^{-4}	1.24	0.76-20.0	3.89×10^{-1}	1.13	0.68-1.87	6.38×10^{-1}	70.42	0.93	0.60-1.42	7.21×10^{-1}
rs6418572	26109128	47kb 5' of *MAGEB18*	0.43	0.29-0.65	5.11×10^{-5}	1.89	0.92-3.87	8.33×10^{-2}	2.14	0.92-4.98	7.79×10^{-2}	87.42	1.15	0.40-3.27	8.00×10^{-1}
rs6603446	115968398	374kb 3' of *CT83*	0.68	0.54-0.85	7.25×10^{-4}	0.97	0.64-1.47	8.82×10^{-1}	1.63	1.00-2.64	4.76×10^{-2}	81.71	0.99	0.60-1.62	9.60×10^{-1}

Table S7. Regulatory annotation of X-chromosme SNPs using HaploReg databases.

Gene	r²	snp	Ref	Alt	AFR	AMR	ASN	EUR	Promoter histone marks	Enhancerhistone marks	DNAse	Proteins bound	Motifs changed
DMD	1.00	rs6631437	T	C	0.51	0.29	0.21	0.18					CDP,Duxl,HNF1
	**1.00**	**rs5927056**	**T**	**G**	**0.59**	**0.3**	**0.21**	**0.17**					**6 altered motifs**
ARHGAP6	0.81	rs149935670	A	G	0.17	0.18	0.19	0.03					HDAC2,Isl2,STAT
	0.82	rs17327753	T	C	0.2	0.21	0.19	0.08		GI			6 altered motifs
	0.82	rs66720805	A	G	0.2	0.21	0.19	0.06		GI	LNG		
	0.80	rs17281066	A	T	0.12	0.17	0.19	0.02		4 tissues	6 tissues		CIZ,Foxp3
	0.80	rs145631805	G	A	0.16	0.18	0.19	0.02		FAT, SKIN			HDAC2,VDR
	0.80	rs57131712	C	T	0.11	0.17	0.19	0.03	STRM, BLD	14 tissues	20 tissues	POL2,PU1	CHD2,E2F,PPAR
	0.82	rs17281073	T	C	0.2	0.21	0.19	0.07	4 tissues	13 tissues	7 tissues		Mef2
	0.81	rs9792742	T	A	0.18	0.18	0.19	0.02		SKIN, GI, BLD	4 tissues		
	0.82	rs5935064	T	C	0.2	0.21	0.19	0.07					
	**1.00**	**rs5933886**	**C**	**T**	**0.79**	**0.46**	**0.24**	**0.51**		**FAT**			**5 altered motifs**
	0.81	rs60514383	T	C	0.19	0.19	0.19	0.03		6 tissues	7 tissues	CTCF,RAD21	7 altered motifs
	1.00	rs5979403	A	C	0.37	0.35	0.23	0.34		6 tissues	SKIN,MUS	CTCF	NRSF,Pax-4
	0.82	rs61295215	G	A	0.2	0.21	0.19	0.06		5 tissues	10 tissues		Gm397
	1.00	rs5978436	G	A	0.35	0.32	0.24	0.31		6 tissues	LNG,OVRY		5 altered motifs
	0.82	rs66989301	T	C	0.2	0.21	0.19	0.07					5 altered motifs
	0.80	rs186178927	C	T	0.13	0.18	0.19	0.03		5 tissues			BCL,NRSF,Sin3Ak-20
	0.81	rs73500862	T	C	0.33	0.2	0.19	0.05		5 tissues	6 tissues	GATA3	LRH1

Table S8. Significant *cis*-eQTL results for SNPs nearby rs5933886 (<±250Kb and r^2> 0.8 in either males or females) in *ARHGAP6* from GTEx databases.

Variant Id	rsid	*P*	NES	Tissue	r^2 in males	r^2 in females
X_11451034_T_C_b37	rs17327753	1.40E-07	0.36	Artery - Aorta	0.82	0.78
X_11451183_A_G_b37	rs66720805	5.60E-06	0.32	Artery - Aorta	0.82	0.78
X_11451183_A_G_b37	rs66720805	4.40E-05	0.17	Cells - Transformed fibroblasts	0.82	0.78
X_11456334_T_C_b37	rs17281073	5.40E-07	0.35	Artery - Aorta	0.81	0.78
X_11456334_T_C_b37	rs17281073	4.80E-05	0.17	Cells - Transformed fibroblasts	0.81	0.78
X_11462392_C_T_b37	rs5933885	4.30E-06	0.17	Testis	NA	NA
X_11462392_C_T_b37	rs5933885	3.80E-05	-0.16	Muscle - Skeletal	NA	NA
X_11462856_G_GACTAC_b37	NA	7.40E-07	0.35	Artery - Aorta	NA	NA
X_11462858_A_T_b37	rs752419032	4.50E-07	0.36	Artery - Aorta	NA	NA
X_11462860_A_G_b37	rs756339827	4.50E-07	0.36	Artery - Aorta	NA	NA
X_11462863_A_ACTTTTG_b37	rs200979100	7.40E-07	0.35	Artery - Aorta	NA	NA
**X_11463381_C_T_b37**	**rs5933886**	**5.85E-03**	**0.13**	**Artery - Aorta**	**1**	**1**
X_11466082_T_C_b37	rs66989301	6.70E-07	0.36	Artery - Aorta	0.81	0.78
X_11466082_T_C_b37	rs66989301	3.40E-05	0.16	Esophagus - Muscularis	0.81	0.78
X_11466082_T_C_b37	rs66989301	5.00E-05	0.17	Cells - Transformed fibroblasts	0.81	0.78
X_11466939_T_A_b37	rs73184360	8.30E-05	-0.42	Muscle - Skeletal	NA	NA
X_11467342_A_G_b37	rs62587962	5.80E-11	-0.31	Muscle - Skeletal	NA	NA
X_11468683_T_C_b37	rs73500862	3.80E-06	0.33	Artery - Aorta	0.81	0.77

NA denotes that SNPs was not covered in current study.

Table S9. Power analysis of association tests in this study.

Marker	Annotation	MAF	*OR*	power in discovery data	power in replication data	power in combined data
**All samples**						
rs5927056	*DMD*	0.29	0.81	0.170	0.028	0.442
rs6641142	299kb 3' of *LOC101928201*	0.15	1.28	0.169	0.024	0.426
rs12860876	*REPS2*	0.37	1.2	0.139	0.021	0.372
rs6528069	43kb 5' of *SMS*	0.46	0.85	0.082	0.013	0.243
rs5949698	430kb 5' of *BRDTP1*	0.17	1.22	0.067	0.010	0.200
rs5910990	108kb 3' of *C1GALT1C1*	0.5	0.86	0.057	0.009	0.176
rs2207942	261kb 5' of *CXorf51A*	0.3	1.18	0.066	0.010	0.198
rs2018094	*FRMPD4*	0.38	0.86	0.046	0.007	0.145
**Males**						
rs6641142	299kb 3' of *LOC101928201*	0.14	1.31	0.107	0.015	0.296
rs5927056	*DMD*	0.3	0.81	0.081	0.013	0.241
rs6629842	42kb 3' of *ZFX*	0.4	1.2	0.065	0.010	0.196
rs12860876	*REPS2*	0.35	1.2	0.058	0.009	0.176
rs5949698	430kb 5' of *BRDTP1*	0.16	1.25	0.049	0.007	0.149
rs4484871	*PTCHD1-AS*	0.15	0.78	0.044	0.008	0.142
rs5910990	108kb 3' of *C1GALT1C1*	0.51	0.85	0.037	0.006	0.117
rs2207942	261kb 5' of *CXorf51A*	0.3	1.19	0.037	0.006	0.116
rs6528069	43kb 5' of *SMS*	0.47	0.85	0.036	0.006	0.114
rs371000	*F9*	0.13	1.25	0.031	0.005	0.097
rs1323329	50kb 3' of *LINC00632*	0.47	0.85	0.036	0.006	0.114
**Females**						
rs6418572	47kb 5' of *MAGEB18*	0.12	0.43	0.433	0.129	0.841
rs10442369	*EFHC2*	0.33	0.63	0.321	0.066	0.703
rs12847598	17kb 5' of *VENTXP1*	0.11	0.46	0.280	0.072	0.676
rs6633192	*ADGRG2*	0.42	1.54	0.394	0.075	0.769
rs12834592	143kb 5' of *NAP1L3*	0.39	1.51	0.320	0.056	0.686
rs5933886	*ARHGAP6*	0.27	0.62	0.266	0.054	0.634
rs4495593	294kb 3' of *VENTXP1*	0.33	0.65	0.240	0.046	0.589
rs5972485	*DMD*	0.21	0.6	0.218	0.045	0.563
rs6603446	374kb 3' of *CT83*	0.49	0.68	0.233	0.041	0.569
rs5935253	*FRMPD4*	0.37	0.66	0.242	0.045	0.590
rs5935567	82kb 3' of *LINC01203*	0.2	0.59	0.226	0.047	0.578
